# A Dietary-Wide Association Study (DWAS) of Environmental Metal Exposure in US Children and Adults

**DOI:** 10.1371/journal.pone.0104768

**Published:** 2014-09-08

**Authors:** Matthew A. Davis, Diane Gilbert-Diamond, Margaret R. Karagas, Zhigang Li, Jason H. Moore, Scott M. Williams, H. Robert Frost

**Affiliations:** 1 Children's Environmental Health and Disease Prevention Research Center at Dartmouth, Hanover, New Hampshire, United States of America; 2 Institute for Quantitative Biomedical Sciences, Dartmouth College, Hanover, New Hampshire, United States of America; 3 University of Michigan School of Nursing, Ann Arbor, Michigan, United States of America; 4 Section of Biostatistics and Epidemiology, Department of Community and Family Medicine, Geisel School of Medicine at Dartmouth, Hanover, New Hampshire, United States of America; 5 Department of Genetics, Geisel School of Medicine at Dartmouth, Hanover, New Hampshire, United States of America; Case Western Reserve University, United States of America

## Abstract

**Background:**

A growing body of evidence suggests that exposure to toxic metals occurs through diet but few studies have comprehensively examined dietary sources of exposure in US populations.

**Purpose:**

Our goal was to perform a novel dietary-wide association study (DWAS) to identify specific dietary sources of lead, cadmium, mercury, and arsenic exposure in US children and adults.

**Methods:**

We combined data from the National Health and Nutrition Examination Survey with data from the US Department of Agriculture's Food Intakes Converted to Retail Commodities Database to examine associations between 49 different foods and environmental metal exposure. Using blood and urinary biomarkers for lead, cadmium, mercury, and arsenic, we compared sources of dietary exposure among children to that of adults.

**Results:**

Diet accounted for more of the variation in mercury and arsenic than lead and cadmium. For instance we estimate 4.5% of the variation of mercury among children and 10.5% among adults is explained by diet. We identified a previously unrecognized association between rice consumption and mercury in a US study population – adjusted for other dietary sources such as seafood, an increase of 10 g/day of rice consumption was associated with a 4.8% (95% CI: 3.6, 5.2) increase in blood mercury concentration. Associations between diet and metal exposure were similar among children and adults, and we recapitulated other known dietary sources of exposure.

**Conclusion:**

Utilizing this combination of data sources, this approach has the potential to identify and monitor dietary sources of metal exposure in the US population.

## Introduction

Human environmental exposure to toxic metals such as lead, mercury, cadmium, and arsenic is associated with a wide array of immediate and long-term health effects. Upon exposure, lead, mercury, and cadmium accumulate in the nervous system, liver, kidneys, and lead accumulates within calcified tissues including bone and teeth [1]–[3]. While arsenic collects in keratin-rich tissues of the integumentary system [4], it is rapidly excreted by the urinary system [5].

The primary target organ for lead is the central and peripheral nervous systems and, as such, exposure is associated with neuropathies and neurobehavioral effects [6]. Infants and children, due to more rapid bone growth and differences in physiology [7], are most vulnerable to the neurotoxic effects of lead, and exposure is associated with a variety of effects on cognition and behavior even at low levels of exposure common in the US and elsewhere [1], [8]. Likewise, methyl-mercury (the predominant form of mercury found in aquatic life [9]) exposure in animal studies is associated with developmental neurotoxic and immune-suppressive effects [2]. Cadmium in particular has a long-half life and is more widely distributed throughout the body but targets the kidney where cadmium accumulates in the epithelial cells of the proximal tubule that can result in reabsorption dysfunction [3], [10]. Recent evidence indicates the potential for adverse health effects from arsenic exposure at the relatively low exposure levels common to populations worldwide, including an increased risk of cancer, cardiovascular and respiratory conditions, and diabetes [11]–[16].

The ingestion of contaminated food has become a well-known source of human exposure to toxic metals [2]. For instance, consumption of seafood has been associated with exposure to considerable amounts of methyl-mercury (especially in the consumption of predatory fish that are higher in the aquatic food chain [17]) and arsenic in adult populations [18], [19]. Second only to smoking [20], dietary sources are considered the most significant route of non-occupational cadmium exposure; cereals, tubers, pulses, and rice are among the most recognized dietary sources of cadmium [21]–[23]. Contamination can occur if crops are grown in tainted soil or treated with metal-contaminated pesticides [2], [24]. For example, arsenic contamination has been reported in rice, grains, fruits, and juices; presumably from both naturally occurring arsenic found in soil as well as use of arsenic-containing pesticides [25]–[28]. The exact mechanism of crop contamination differs based on both the specific environmental metal and crop. For instance, arsenic accumulates in rice [29] through the silicon transport system [30] because, to the rice plant, arsenous acid (the major form of arsenic in flooded rice paddies) is indistinguishable from silicic acid.

Despite considerable interest in identifying dietary sources of toxic metal exposure, environmental health studies typically examine only a few dietary sources at one time and broader approaches rely on less rigorous study designs such as market basket surveys. To our knowledge previous studies have not comprehensively examined dietary sources of environmental metal exposure in a large US study population. Examining associations between the various foods (particularly specific crops) and human exposure to metals could not only uncover sources of exposure, but could also potentially identify protective foods – foods whose nutritional composition might inhibit absorption of toxic metals.

Following in the footsteps of global investigations of associations such as genome-wide association studies (GWAS) [31], [32] and the more recent environmental/exposure-wide association studies (EWAS) [33], [34], we examined associations between 49 different foods and biomarkers of lead, mercury, cadmium, and arsenic exposure. To do so, we combined data from the National Health and Nutrition Examination Survey (NHANES) and the US Department of Agriculture's Food Intakes Converted to Retail Commodities Database (FICRCD) [35] that estimates the total food commodity intake for the individual foods reported by NHANES. As dietary patterns and metabolism differ according to age [36], we examined sources of dietary exposure to toxic metals among children (age <18 years) and adults (age ≥18 years) separately.

## Methods and Materials

### Study population

The NHANES is a nationally-representative, multi-stage random survey of the non-institutionalized US population that is conducted by the National Center for Health Statistics that gathers detailed information on diet and health from in-person interviews. NHANES study participants also undergo a rigorous clinical examination that includes the collection of blood and urine specimens. For this study, we used data from the NHANES demographics, in-person first day dietary questionnaire, laboratory, physical examination, and health questionnaire files. As our study used publically available and de-identified data, it was determined to be exempt from institutional board review by Dartmouth College's Committee for the Protection of Human Subjects.

**Figure 1:**
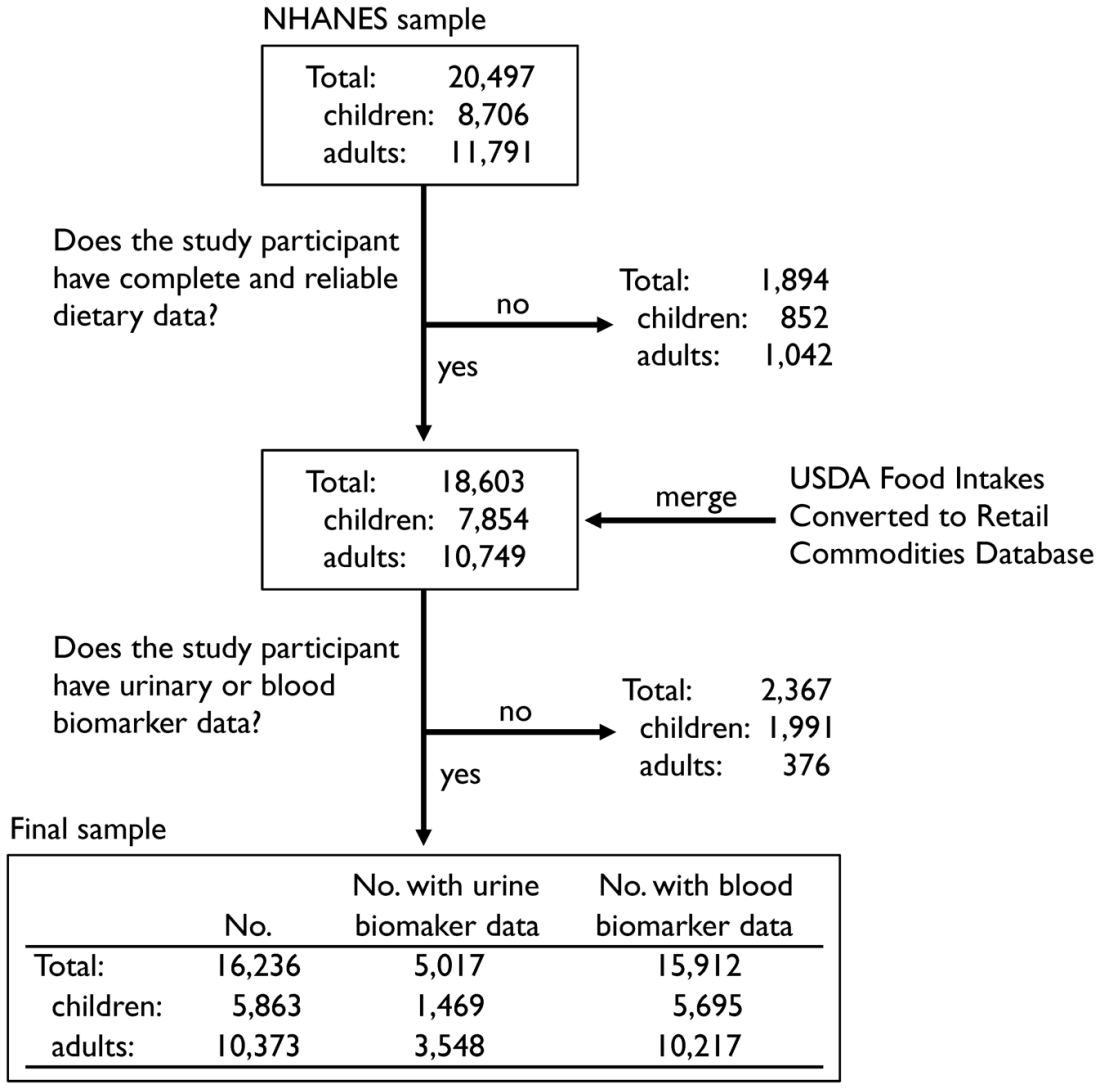
Flow diagram of inclusion for study participants and data sources. Abbreviations: NHANES, National Health and Nutrition Examination Survey; USDA, United States Department of Agriculture.

We analyzed data from all study participants in the 2005–06 and 2007–08 NHANES surveys. During this period, a total of 20,497 individuals participated in the NHANES surveys (Figure 1) and the unweighted overall response rate was 77.4% and 75.4% for the 2005–06 and 2007–08 surveys respectively. NHANES study participants one year and older were eligible for blood lead, mercury, and cadmium laboratory analysis and approximately one-third of the participants were randomly selected for urinary biomarkers. For children (age <18 years), urinary laboratory analyses were restricted to ages between 6 to 17 years.

### Biomarker measurements

After collection at an NHANES mobile examination site, whole blood specimens are stored frozen (−20°C) and transported to the National Center for Environmental Health (NCEH) at the Centers for Disease Control and Prevention for laboratory analysis. Diluted whole blood cadmium, lead, and mercury concentrations were determined using a quadruple inductively coupled plasma dynamic reaction cell mass spectrometry (ICP-DRC-MS) on a PerkinElmer ELAN DRC series (PerkinElmer Instruments Headquarters, Waltham, Massachusetts, USA). This laboratory analysis is an extension of ICP-MS that utilizes dynamic reaction technology to minimize argon-based interference. The interassay coefficient of variation (CV) across lots for blood lead, cadmium, mercury, and inorganic mercury ranged from 3.4 to 21.4% in the 2005–06 and 2007–08 NHANES surveys. The limit of detection for the various blood metals ranged from 0.20 to 0.40 µg/L.

For NHANES, urine was collected in containers from participants and shipped on dry ice (also frozen at −20°C) to the NCEH. At NCEH, urine samples were stored frozen and analyzed within three weeks of collection following standardized protocols. Urinary lead, cadmium, mercury, and total arsenic were also measured using inductively ICP-DRC-MS on a PerkinElmer ELAN DRC II or ELAN 6100 DRC Plus (PerkinElmer Headquarters, Waltham, Massachusetts, USA). Arsenic species and metabolites [arsenous acid, arsenic acid, monomethylarsonic acid (MMA), dimethylarsinic acid (DMA), arsenobetaine, and arsenocholine] were measured using a combination of high performance liquid chromatography and ICP-DRC-MS on a PerkinElmer Series 200 Pump and a PerkinElmer ELAN DRC II respectively (PerkinElmer Headquarters, Waltham, Massachusetts, USA).

The interassay CV among urinary lead, cadmium, total mercury, and total arsenic ranged from 2.3 to 19.9% and among the arsenic species and metabolites between 3.2 to 9.9%. The detection limit among lead, cadmium, total mercury, and total arsenic ranged from 0.04 to 0.74 µg/L. The detection limits for arsenous acid, arsenic acid, MMA, DMA, arsenobetaine, and arsenocholine were 1.20, 1.00, 0.64, 1.70, 0.40, and 0.59 µg/L respectively. Urinary concentration of arsenic is regarded as a valid measure of recent exposure, especially compared to methods that rely on models of exposure from dietary information [5].

### 24-hour dietary assessment

The in-person dietary questionnaire of NHANES collects detailed information on the study participant's diet for the 24-hour period proceeding the clinical and biomarker measurements and is a validated assessment of dietary consumption [37]. During the physical examination, NHANES participants are asked to recall everything they ate and drank in the previous day (24 hours) and trained NHANES personnel use these data to generate estimates of serving size. For children under the age of 12, the dietary component is conducted with the assistance of a proxy (i.e. a parent or other caregiver) and for children between the ages 12 and 17 the survey is administered without the assistance of a proxy.

We used the USDA FICRCD for the corresponding NHANES years to convert the specific serving sizes reported to be consumed during the in-person 24-hour recall period into continuous measures of different food commodities [38]. The FICRCD provides conversion data to estimate the total content of food commodities such as tomatoes, beans, rice, beef etc. in each item with a USDA Food Code reported in NHANES data (e.g., the amount of beans or rice within a burrito). We estimated the total amount in grams of the various food servings consumed by each participant by multiplying the quantity of each food serving reported to be consumed during the 24-hour recall period by the FICRCD estimate of content (dry grams of food commodity per 100 grams of food serving) of the USDA Food Code, then summing across all foods consumed during the 24-hour recall period (Figure S1). As we were interested in identifying specific sources of dietary metal exposure we restricted our analyses to non-aggregate food commodities (i.e. excluded aggregated food groups such as “total diary” and “total meat” from our analyses). However, for the food commodity for eggs we combined the two reported measures “eggs with shell” and “eggs no shell”. We specifically retained separate sources of fruit (e.g. “apples” versus “apple from juice”) to examine potential differences in exposure that may occur from processing. Therefore, our final analyses consisted of 49 separate foods.

### Other data

We also extracted data on sociodemographic characteristics (age, sex, race/ethnicity, marital status, educational attainment, and measures of socio-economic status), health status (self-reported health status and body mass index [BMI]), and information on other exposures (exposure to cigarette smoke, age of home, and drinking water source). We estimated the percentages of the population that were normal weight, overweight, and obese. To do so, for children we calculated the age- and sex- specific BMI Z-score using the 2006 growth curves from World Health Organization [39] for children five years and under. For children between the ages five and 18, we used the 2000 growth curves from the US Centers for Disease Control and Prevention [40] to calculate age- and sex- specific BMI Z-score. Based on the age- and sex- specific BMI Z-scores, children were classified as either normal weight (<85^th^ percentile), overweight (≥85^th^ to 95^th^ percentile), or obese (≥95^th^ percentile). Adults were also classified as normal weight, overweight, or obese based on a body mass index of <25.0 kg/m^2^; ≥25.0 kg/m^2^ to 30.0 kg/m^2^; and ≥30.0 kg/m^2^ respectively. We classified race/ethnicity as “Non-Hispanic White”, “Non-Hispanic Black”, “Hispanic or Mexican American” and “Other, multiple race/ethnicity.” As a surrogate measure of potential occupational exposure to the four metals, we used self-reported employment status and reported hours working in the prior week to classify participants as not working (zero hours), part time (one to 40 hours), and full time (40 hours or more).

To account for lead exposure by contact with paint containing lead, we collapsed NHANES participant's self-reported age of home into those built prior to and after 1978 (the year lead paint was banned [41]). Because cigarette smoke is a source of cadmium and arsenic exposure [42], [43], we used serum cotinine to account for active exposure to cigarette smoke. The NHANES measures serum cotinine using an isotope-dilution HPLC/atmospheric pressure chemical ionization mass spectrometry method.

In the US, arsenic exposure through drinking water is primarily found in private unregulated water systems and lead exposure can occur through drinking water contact with lead pipes [44]. Therefore we used the reported water consumed in the 24-hour dietary assessment period and self-reported drinking water source to create a measure of public (using a community water source) and private (defined as either a well, spring, or cistern water source) water consumption measured in grams per day.

In our analyses of urinary biomarkers, we used urinary creatinine to account for urinary dilution. In the 2005–06 NHANES survey, urinary creatinine was measured on a Beckman Synchron CX3 clinical analyzer (Beckman Instruments Inc., Brea, California, USA) using a Jaffe reaction and in the 2007–2008 survey it was measured on a Roche Modular P Chemistry Analyzer (Roche Diagnostics, Indianapolis, Indiana, USA) using an enzymatic method. Therefore, we adjusted 2005–06 urinary creatinine measurements to 2007–08 equivalents using a piecewise regression supplied by the NHANES [45].

### Statistical analyses

Blood mercury level is an estimate of exposure to organic mercury (approximately 90% of blood mercury is methyl-mercury) and essentially all mercury in urine is inorganic [19]. We evaluated organic and inorganic forms of arsenic separately (i.e. blood versus urinary mercury), due to their differing toxicity [46]. For urinary arsenic, due to uncertain or negligible health impacts of arsenobetaine and arsenocholine exposure, we subtracted these components from the total urinary arsenic concentrations – thus our final definition of total urinary arsenic included arsenous acid, arsenic acid, MMA, and DMA [38], [47]. For the 56 participants (1.1% of participants with urinary arsenic assessment) for whom the combination of arsenobetaine and arsenocholine exceeded the estimate of total arsenic reported by NHANES (presumably due to laboratory imprecision) we assigned a total arsenic value of zero.

We used an adjusted R-squared measure as our primary estimate of the overall contribution of diet to variation in the four biomarkers. We used several different approaches to examine the relationship between the reported consumption of specific foods and biomarker metal concentrations. First, we used Spearman correlation coefficients to explore relationships between the different foods and blood and urine biomarker concentrations. We then used linear models to adjust for confounding factors including all other foods in our analyses. For interpretation, our linear models estimate the effect of an increase of 10 grams per day of the various foods. We log_10_-transformed all biomarker variables to improve model fit and help normalize residuals and exponentiated all coefficients to represent the % change in biomarker concentration based on an increase of 10 grams per day for each food. Our linear models were based on complete case analysis. The analysis of NHANES data typically utilizes complex survey design methods in order to generate national estimates. As some of the analytic techniques used in our analyses are not yet fully developed in complex survey design methods (e.g., adjusted R-squared) and to maintain internal consistency, none of our analyses used complex survey methods.

To correct for multiple hypothesis testing across the various biomarkers for each food, we corrected for the false discovery rate [48]. For validation of potential associations between foods and environmental metal exposure, we also constructed a validation dataset (referred to herein as a validation study population) from 2003–04 NHANES and FICRCD data using the identical methods to that as our primary analytic dataset.

We used Stata version 13.0 (StatCorp, College Station, Texas, USA) for the management of NHANES data files and R (R Foundation for Statistical Computing, Vienna, Austria) for all statistical analyses.

## Results

### Characteristics of study participants

In the 2005–06 and 2007–08 NHANES surveys we identified 16,236 study participants (5,863 children and 10,373 adults) with complete dietary data and either a blood or urinary biomarker for lead, cadmium, mercury, or arsenic (Figure 1).

Among both children and adults the study population was approximately evenly distributed by sex. As expected, because NHANES oversamples minorities, racial/ethnic minorities were more heavily represented (e.g., 30.6% of children were of Hispanic or Mexican American background versus 29.1% who were Non-Hispanic White) in the study population (Table S1). Of the adults in our study, 46.0% reported not working in the past week, whereas 17.2% worked between one and 40 hours, and 36.7% reported 40 or more hours. The majority of study participants had serum cotinine levels in excess of 0.015 µg/L, and approximately 40% (35.8% of children and 43.3% of adults) were identified as residing in a house built before 1978. We classified 19.8% of children and 34.4% of adults as being obese.

### Dietary sources of environmental metal exposure

#### Lead

Diet explained a greater amount of blood lead variation in children than in adults (2.9% of the variation in blood lead was explained by diet in children versus 1.6% in adults) (Table 1). While particularly in children a variety of foods were associated with blood lead concentration (e.g., for both whole milk and apple r_s_ = 0.15), consumption of rice among children was weakly associated with an increase in blood lead concentration in adjusted models; 10 g/day of dried rice was associated with a 1.6% (95% CI: 0.7, 2.5) increase in blood lead concentration. However, this association was not observed in our validation study population. Interestingly, consumption of oat flour was associated with a lower blood lead concentration in adults (a 10 g/day increase in oat flour was associated with a 1.8% decrease in blood lead concentration). Furthermore, an even stronger association (a 3.5% decrease per 10 g/day increase) between oat flour and lower blood lead concentration in adults was observed in our validation study population (Figure S2).

**Table 1:** Amount of variation of lead, cadmium, mercury, and arsenic exposure explained by diet among children versus adults.

	Children			Adults		
	Adjusted R-squared		% explained by Diet	Adjusted R-squared		% explained by Diet
Biomarker	Base model^a^	Base model + Diet^c^		Base model^a^^,^ b	Base model + Diet^c^	
Blood						
Lead	0.17	0.20	2.9%	0.37	0.39	1.6%
Cadmium	0.36	0.38	1.4%	0.35	0.36	0.6%
Mercury	0.02	0.06	4.5%	0.04	0.14	10.5%
Urine^d^						
Lead	0.34	0.35	0.5%	0.43	0.44	1.3%
Cadmium	0.41	0.41	−0.2%	0.51	0.51	0.3%
Mercury	0.17	0.16	0.0%	0.24	0.27	2.5%
Total arsenic^e^	0.31	0.39	8.5%	0.32	0.43	11.5%
Arsenobetaine	0.01	0.16	14.8%	0.04	0.17	12.8%
DMA	0.29	0.40	11.5%	0.31	0.45	13.7%
MMA	0.20	0.23	2.6%	0.20	0.26	5.4%

Abbreviations: DMA, dimethylarsinic acid; MMA, monomethylarsonic acid.

a : base model covariates include age (continuous, years), sex, body mass index (continuous, for children Z-score and kg/m^2^ for adults), serum cotinine (continuous, µg/L), and age of home (built before 1978 versus after 1978).

b : further adjusted for employment status (not working versus part- or full-time).

c : dietary data includes the 49 foods and water variables as independent variables (10 g/day).

d : further adjusted for urinary creatinine (continuous, mg/dL) to account for urinary dilution.

e : excludes arsenobetaine and arsenocholine.

#### Cadmium

Among the four metals we examined, diet explained the least amount of detectable variation in cadmium exposure. Diet explained only 1.4% in children and 0.6% in adults of the variation in blood cadmium concentration (a short term biomarker) and less than 1.0% of variation in urinary cadmium concentration (a longer-term exposure biomarker) (Table 1).

**Figure 2:**
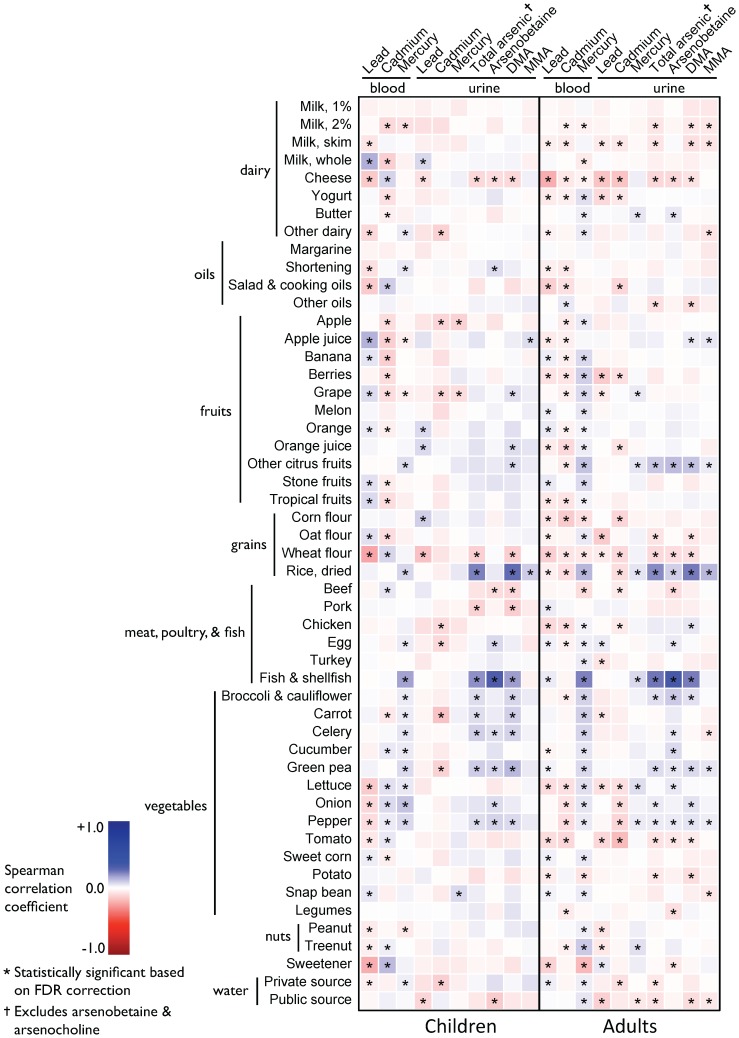
Spearman correlation coefficients between grams of food per day and lead cadmium, mercury, and arsenic biomarker concentrations among children versus adults. Abbreviations: DMA, dimethylarsinic acid; MMA, monomethylarsonic acid; FDR, false discovery rate.

**Figure 3:**
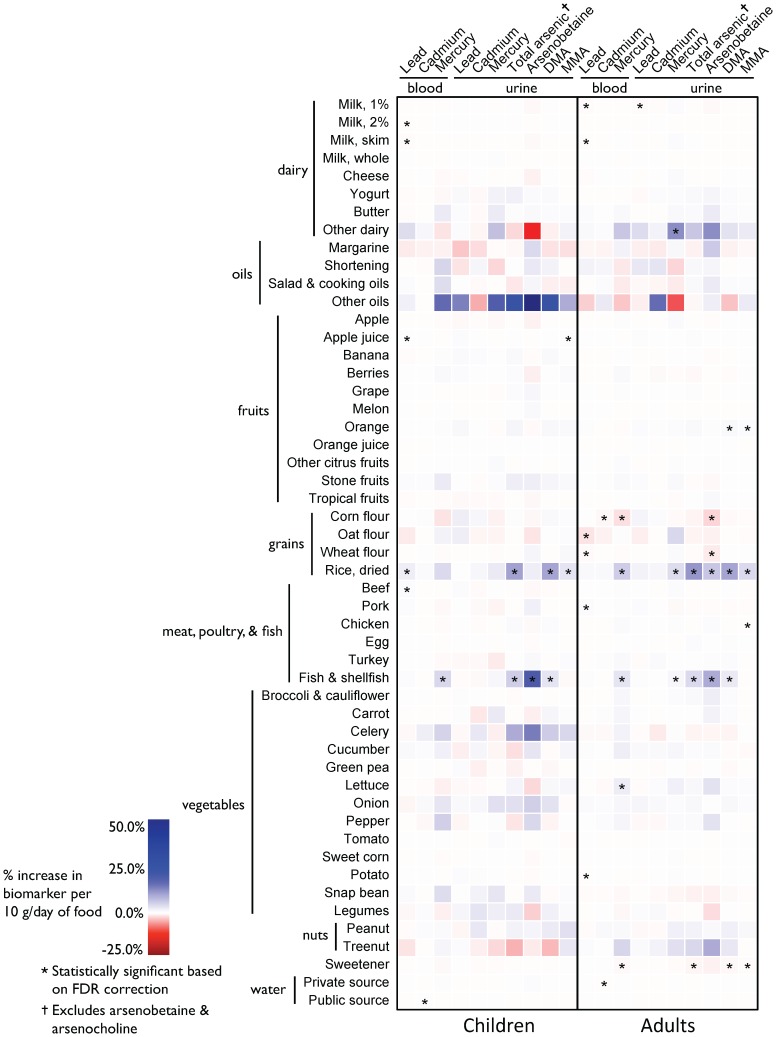
Percent change in lead, cadmium, mercury, and arsenic biomarker concentrations based on an increase of 10 grams of food per day among children versus adults. All models adjusted for age (continuous, years), sex, body mass index (continuous, Z-score for children and kg/m^2^ for adults), serum cotinine (continuous, µg/L), and age of home (built before 1978 versus after 1978) and all other dietary sources in table (continuous, 10 g/day). Urinary biomarker models further adjusted for urinary creatinine (continuous, mg/dL) to account for urinary dilution and models restricted to adults also adjusted for employment status (not working versus full- or part-time). Abbreviations: DMA, dimethylarsinic acid; MMA, monomethylarsonic acid; FDR, false discovery rate.

Although weak positive associations between cheese, salad & cooking oils, and some vegetables were associated with blood cadmium concentration in children and negative associations were found between foods and blood cadmium levels in adults, few associations persisted in adjusted models and in our validation study population (Figure 2 versus Figure 3).

#### Mercury

We found stronger associations between diet and blood mercury concentration (a biomarker of primarily organic mercury) compared to lead and cadmium. Diet accounted for 4.5% of the variation in blood mercury concentration in children and 10.5% in adults.

In unadjusted and adjusted analyses, both seafood and rice consumption were associated with increases in blood mercury concentration. In adults, the Spearman correlation coefficient between fish & seafood and blood mercury concentration was 0.24 and the correlation between rice and blood mercury concentration was 0.14 (Figure 2). In models adjusted for other dietary sources, water, and sociodemographic characteristics, an increase of 10 g/day of rice and seafood consumption was associated with a 4.8% (95% CI: 3.6, 5.2) and 2.5% (95% CI: 2.2, 2.8) increase in blood mercury concentration respectively in adults (Figure 3). Both these associations were reproduced in our validation study population (Figure S2).

#### Arsenic

Diet had the strongest relationship to arsenic exposure among the four different metals we examined, and it accounted for 8.5% of the variation in total urinary arsenic (excluding arsenobetaine and arsenocholine) among children and 11.5% among adults (Table 1). When examining specific arsenic species, diet explained an even greater proportion of the variability in arsenic exposure (e.g., diet explained 13.7% of the variation in DMA in adults).

We found particularly strong associations between consumption of both rice and seafood and urinary arsenic concentration among both children and adults (Figure 2 and Figure 3). In adults, an increase of 10 g/day of dried rice was associated with an increase of 9.6% (95% CI: 8.2, 11.1) increase in total urinary arsenic and an 8.6% (95% CI: 7.6, 9.6) increase in DMA in adults. Similarly, an increase of 10 g/day of seafood in adults was associated with an increase of 3.4% (95% CI: 2.9, 3.9) in total urinary arsenic; similar associations were found in children as well. Although attenuated, we also found associations between rice consumption and MMA in both children and adults. All the associations between both rice and seafood and urinary arsenic concentration (both total urinary arsenic and DMA) were reproduced in our validation study population (Figure S2).

## Discussion

To our knowledge, this is the first large-scale DWAS of environmental exposure to lead, cadmium, mercury, and arsenic. Using a combination of data from the NHANES and the USDA FICRCD, we were able to recapitulate known associations between dietary sources of metal exposure as well as uncover new potential sources of metal exposure. A particular strength of our approach to estimating intake of specific food types is the ability to adjust for other dietary sources to identify independent associations.

There is considerable interest in examining associations across many environmental factors including the contribution of diet and nutrient intake to human disease. Previous investigations have used NHANES data to perform nutrient-wide association studies (NWAS) and have examined associations between a wide array of nutrients and serum lipid levels [49] and blood pressure [50]. These studies are particularly well suited to disentangle biological underpinnings of disease (i.e. how different nutrients may affect health states); however, the strength of our approach lies in the ability to identify sources of metal exposure from specific foods. Up to this point, quantifying patterns of dietary consumption has been a challenge among researchers. While our analyses focused on identify sources of environmental metal exposure, combining NHANES and FICRCD data would also allow researchers the ability to explore associations between specific dietary profiles and health outcomes. Furthermore, because FICRCD data include information for a wide variety of foods, it may reduce bias in estimated associations by taking into account all dietary sources in statistical models. Lastly, our study begins to fill a gap in understanding the connection between environmental and internal measures of exposure.

As previously demonstrated in US populations, we found relationships between rice and seafood consumption with arsenic exposure [18], [38], [47], [51] and determined that dietary patterns of metal exposure were similar between children and adults. Overall, we identified specific dietary sources of arsenic, mercury, and lead and detected a relative paucity of dietary exposure to cadmium in this study population. Because of the richness of the dietary data, we also uncovered potentially new dietary associations with metal exposures in this US population. In adjusted models, and validated in an independent study population, we found that rice consumption was associated with mercury exposure adults. This finding has particular relevance to future US safe food practices and may warrant further investigation. Previous studies have examined contaminated rice in China. In these studies, while most rice samples were below maximum allowable concentrations for toxic metals – estimates of lead for children and mercury for adults exceeded safe limits in some locales [52]–[55]. Interestingly, we also found evidence in our primary and validation study populations that certain foods may reduce exposure to toxic metals. For example, we found inverse associations between blood lead and oat flour suggesting that these foods (potentially due to their nutritional properties) may interfere with lead absorption.

### Limitations

Our study has several limitations that must be acknowledged. First, our estimates of food consumption are based on self-report and therefore may be subject to misclassification. Despite the NHANES being known as reliable source of dietary data [37], due to measurement error our results are more likely biased towards the null. Second, as we did not apply the NHANES complex survey design methods to our analyses, our results are not necessarily generalizable to the entire US population. The NHANES sampling frame is the non-institutionalized population (i.e. excludes those in the military, nursing homes, etc.) and over samples minorities, thus our results should not be interpreted as representing the entire US population. However, while our estimates may not reflect the US, considering the more diverse sample, over-representation of minority groups may also be considered a strength. Third, because of wide variation in food growing practices and geology (e.g., there exists wide variation in arsenic concentration in rice due to both cultivation and species [56]), we are not able to draw conclusions regarding the specific amount of lead, cadmium, mercury, and arsenic exposure from the foods we examined.

Nonetheless, using a novel combination of data sources and methods from both informatics and epidemiology, we were able to observe known associations and identify potential new associations in a large US study population. This study provides an important demonstration of a DWAS of environmental metal exposures.

## Supporting Information

Figure S1 **National Health and Nutrition Examination Survey (2005–06 and 2007–08 surveys) first day dietary recall data converted to food commodities according to study participant sequence number.**
(CSV)

Figure S2 **Percent change in lead, cadmium, mercury, and arsenic biomarker concentrations based on an increase of 10 grams of food per day among children versus adults in validation study population.** All models adjusted for age (continuous, years), sex, body mass index (continuous, Z-score for children and kg/m^2^ for adults), serum cotinine (continuous, µg/L), and age of home (built before 1978 versus after 1978) and all other dietary sources in table (continuous, 10 g/day). Urinary biomarker models further adjusted for urinary creatinine (continuous, mg/dL) to account for urinary dilution and models restricted to adults also adjusted for employment status (not working versus full- or part-time). Abbreviations: DMA, dimethylarsinic acid; MMA, monomethylarsonic acid; FDR, false discovery rate.
(TIFF)

Table S1 **Characteristics of children versus adult study participants.**
(PDF)
