# Supplementary figures and images for: A Dietary-Wide Association Study (DWAS) of Environmental Metal Exposure in US Children and Adults
Source: PLoS One. 2014 Sep 8;9(9):e104768. doi: 10.1371/journal.pone.0104768 (PMC4157769; doi:10.1371/journal.pone.0104768)

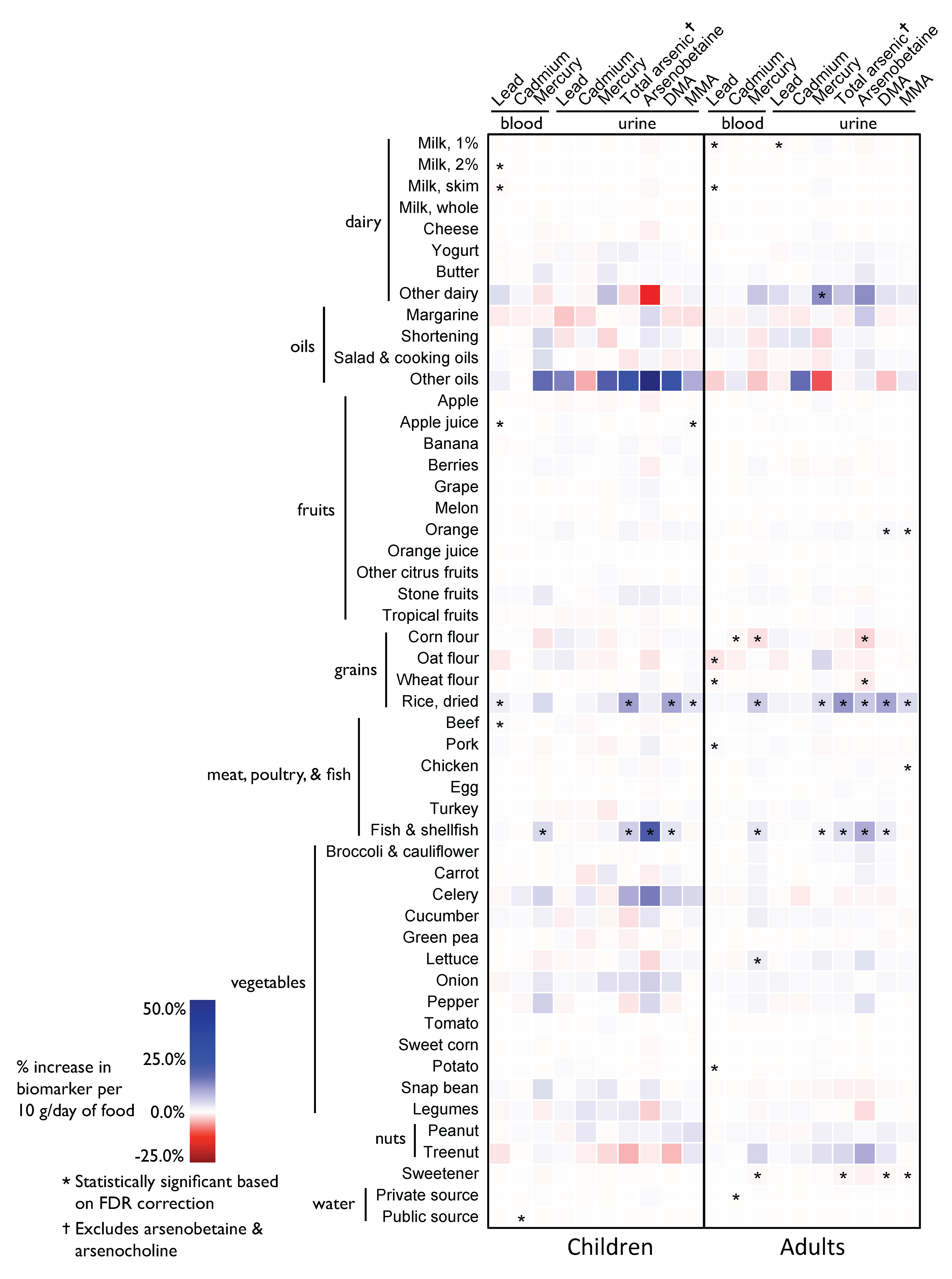

Supplement: Figure S2 — Percent change in lead, cadmium, mercury, and arsenic biomarker concentrations based on an increase of 10 grams of food per day among children versus adults in validation study population. All models adjusted for age (continuous, years), sex, body mass index (continuous, Z-score for children and kg/m2 for adults), serum cotinine (continuous, µg/L), and age of home (built before 1978 versus after 1978) and all other dietary sources in table (continuous, 10 g/day). Urinary biomarker models further adjusted for urinary creatinine (continuous, mg/dL) to account for urinary dilution and models restricted to adults also adjusted for employment status (not working versus full- or part-time). Abbreviations: DMA, dimethylarsinic acid; MMA, monomethylarsonic acid; FDR, false discovery rate. (TIFF) [file pone.0104768.s002.tiff]
