# Supplementary material for: A Dietary-Wide Association Study (DWAS) of Environmental Metal Exposure in US Children and Adults
Source: PLoS One. 2014 Sep 8;9(9):e104768. doi: 10.1371/journal.pone.0104768 (PMC4157769; doi:10.1371/journal.pone.0104768)
Supplement: Table S1 — Characteristics of children versus adult study participants. (PDF) [file pone.0104768.s003.pdf]

Table S1. Characteristics of children versus adult study participants.

Characteristic	No. (%)		
	Total	Children	Adults
Total study participants, sample	16,236	5,863	10,373
Sex			
Male	8,024 (49.4)	2,974 (50.7)	5,050 (48.7)
Female	8,212 (50.6)	2,889 (49.3)	5,323 (51.3)
Race/ethnicity			
Non-Hispanic White	6,613 (40.7)	1,704 (29.1)	4,909 (47.3)
Non-Hispanic Black	3,869 (23.8)	1,604 (27.4)	2,265 (21.8)
Hispanic or Mexican American	3,806 (23.4)	1,794 (30.6)	2,012 (19.4)
Other, multiple	1,948 (12.0)	761 (13.0)	1,187 (11.4)
Marital Status			
Married or living with partner	6,022 (37.0)	18 (0.3)	6,004 (57.9)
Widowed, divorced, or separated	2,147 (13.2)	4 (0.1)	2,143 (20.7)
Never married	2,917 (18.0)	947 (16.2)	1,970 (19.0)
Unknown	5,150 (31.7)	4,894 (83.5)	256 (2.5)
Educational attainment			
Highschool or less	5,127 (31.6)	--	5,127 (49.4)
Some college	2,596 (16.0)	--	2,596 (25.0)
College or more	1,869 (11.5)	--	1,869 (18.0)
Unknown	6,644 (40.9)	5,863 (100.0)	781 (7.5)
Annual family income level, US$			
< $35,000	7,546 (46.5)	2,763 (47.1)	4,783 (46.1)
$35,000 to < $75,000	4,551 (28.0)	1,629 (27.8)	2,922 (28.2)
≥ $75,000	3,281 (20.2)	1,222 (20.8)	2,059 (19.9)
Unknown	858 (5.3)	249 (4.3)	609 (5.9)
Employment past week, hours			
Not working, 0	5,328 (32.8)	557 (9.5)	4,771 (46.0)
Part-time, 1 to 40	1,985 (12.2)	200 (3.4)	1,785 (17.2)
Full-time, > 40	3,835 (23.6)	24 (0.4)	3,811 (36.7)
Unknown	5,088 (31.3)	5,082 (86.7)	6 (0.1)
Serum cotinine, µg/L			
< 0.015	2,606 (16.0)	864 (14.7)	1,742 (16.8)
0.015 to < 10.0	9,373 (57.7)	3,603 (61.5)	5,770 (55.6)
≥ 10.0	2,829 (17.4)	188 (3.2)	2,641 (25.5)
Unknown	1,428 (8.8)	1,208 (20.6)	220 (2.1)

(continued)

Supplemental Material, Table S1. Continued from previous page.

Characteristic	No. (%) Total	Children	Adults
Age of home, year built			
Prior to 1978	6,586 (40.6)	2,157 (36.8)	3,765 (36.3)
1978 or later	5,922 (36.5)	2,098 (35.8)	4,488 (43.3)
Unknown	3,728 (23.0)	1,608 (27.4)	2,120 (20.4)
Body mass index†			
Normal weight	7,011 (43.2)	3,806 (64.9)	3,205 (30.9)
Overweight	4,353 (26.8)	898 (15.3)	3,455 (33.3)
Obese	4,731 (29.1)	1,159 (19.8)	3,572 (34.4)
Unknown	141 (0.9)	--	141 (1.4)
Self-reported health status			
Excellent, very good, or good	9,611 (59.2)	1,936 (33.0)	7,675 (74.0)
Fair or poor	2,432 (15.0)	244 (4.2)	2,188 (21.1)
Unknown	4,193 (25.8)	3,683 (62.8)	510 (4.9)

† For children based on < 85th percentile (normal), 85th to < 95th percentile (overweight) and ≥ 95th percentile (obese) and for adults < 25.0 kg/m^2 (normal), ≥ 25.0 to 30.0 kg/m^2 (overweight), and ≥ 30.0 kg/m^2 (obese).
